# Generation of a Homozygous Transgenic Rat Strain Stably Expressing a Calcium Sensor Protein for Direct Examination of Calcium Signaling

**DOI:** 10.1038/srep12645

**Published:** 2015-08-03

**Authors:** Kornélia Szebényi, András Füredi, Orsolya Kolacsek, Enikő Pergel, Zsuzsanna Bősze, Balázs Bender, Péter Vajdovich, József Tóvári, László Homolya, Gergely Szakács, László Héja, Ágnes Enyedi, Balázs Sarkadi, Ágota Apáti, Tamás I. Orbán

**Affiliations:** 1 Institute of Enzymology, Research Centre for Natural Sciences, Hungarian Academy of Sciences, Budapest, Hungary; 2 NARIC-ABC, Gödöllő, Hungary; 3 ImmunoGenes Ltd., Budakeszi, Hungary; 4 Department of Clinical Pathology and Oncology, Faculty of Veterinary Science, Szent István University, Budapest, Hungary; 5 Department of Experimental Pharmacology, National Institute of Oncology, Budapest, Hungary; 6 Institute of Organic Chemistry, Research Centre for Natural Sciences, Hungarian Academy of Sciences, Budapest, Hungary; 7 2^nd^ Institute of Pathology, Semmelweis University, Budapest, Hungary; 8 Department of Biophysics and Radiation Biology, Semmelweis University, Budapest, Hungary and MTA-SE Molecular Biophysics Research Group, Budapest, Hungary

## Abstract

In drug discovery, prediction of selectivity and toxicity require the evaluation of cellular calcium homeostasis. The rat is a preferred laboratory animal for pharmacology and toxicology studies, while currently no calcium indicator protein expressing rat model is available. We established a transgenic rat strain stably expressing the GCaMP2 fluorescent calcium sensor by a transposon-based methodology. Zygotes were co-injected with mRNA of transposase and a CAG-GCaMP2 expressing construct, and animals with one transgene copy were pre-selected by measuring fluorescence in blood cells. A homozygous rat strain was generated with high sensor protein expression in the heart, kidney, liver, and blood cells. No pathological alterations were found in these animals, and fluorescence measurements in cardiac tissue slices and primary cultures demonstrated the applicability of this system for studying calcium signaling. We show here that the GCaMP2 expressing rat cardiomyocytes allow the prediction of cardiotoxic drug side-effects, and provide evidence for the role of Na^+^/Ca^2+^ exchanger and its beneficial pharmacological modulation in cardiac reperfusion. Our data indicate that drug-induced alterations and pathological processes can be followed by using this rat model, suggesting that transgenic rats expressing a calcium-sensitive protein provide a valuable system for pharmacological and toxicological studies.

The importance of proper calcium homeostasis and signaling from the cellular to the complex organ levels is well appreciated: both in physiological and pathological processes cellular free calcium plays a major role^1^. Disruption of the calcium homeostasis by pharmacological agents or pathological conditions correlate with various conditions, including prolonged QT intervals and arrhythmias in the heart^2^,^3^, or ischemic kidney injuries resulting in poor outcome for kidney transplantations^4^. In fact, several drugs with various mechanisms of action had to be withdrawn from the market because of side effects caused by disruption of the calcium homeostasis, including Clobutinol, a cough suppressant^5^, Dofetilide, an antiarrhythmic agent^6^, Grepafloxacin and Sparfloxacin, antibacterial agents^7^, Terfenadine, an antihistamine^8^, or Terodiline, a spasmolytic agent^9^. All these findings suggest that in the process of drug discovery an early prediction of toxicity requires the direct examination of the drug effects on cellular calcium homeostasis and signaling in different target tissues, especially in the heart.

Animals stably expressing high-sensitivity cellular calcium indicator proteins are best suitable for direct examination of calcium signaling events in cells, tissues and organs as well. A well-established genetically engineered calcium sensor protein is the GCaMP2, containing a calmodulin-based sensor and a GFP-based fluorescent protein, which can be directly used to determine the changes in cellular calcium concentration^10^. The GCaMP2 protein has already been applied in tissue preparations and in transgenic mice^11^,^12^,^13^,^14^, as well as in human pluripotent stem cells^15^, allowing calcium imaging without additional manipulation. However, a calcium sensor expressing rat model has not been available yet.

Several methods are available for the transgenesis of rats, however, transposase-catalyzed gene delivery provides advantages, such as increased efficiency of chromosomal integration and single-copy insertion, while the system is less prone to genetic mosaicism and gene silencing than lentiviral gene delivery^16^. It has also been documented that the SB100X-mediated transgene integration allows the generation of transgenic lines with tissue-specific expression patterns, specified by selected promoter elements^17^.

In the present work we have generated transgenic laboratory rats expressing the fluorescent calcium sensor protein GCaMP2. In order to achieve high-level expression in cardiac tissues, GCaMP2 expression in our model system is driven by a CAG promoter variant proved to be highly active in human embryonic stem cell-derived cardiomyocytes^18^. Additionally to cardiac tissues, characterization of homozygous CAG-GCaMP2 rats demonstrated appreciable GCaMP2 expression in kidney cortex, liver, and blood cells. CAG promoter specific GCaMP2 expression in blood cells allowed the development of a non-invasive, combined approach of genetic and phenotypic selection, yielding rat strains with high sensor protein expression, in spite of a mono-allelic transgene incorporation.

To validate the applicability of this model system in physiological and pharmacological studies, we used *ex vivo* and *in vitro* cardiomyocyte preparations to examine the effects of various ligands and potential drugs, such as the antimalarial agent, mefloquine, reported to disrupt the calcium homeostasis of heart tissue^19^; terodiline, causing prolongation of the QT interval and cardiac arrhythmia^20^; and terfenadine, known to prolong the QT interval through inhibition of the delayed rectifier potassium current of isolated rat ventricular myocytes^21^. Moreover, we examined the function of the Na^+^/Ca^2+^ exchanger (NCX) by using an *in vitro* cellular hypoxia-reperfusion model, and found a rapid rise in cellular calcium during reoxygenation, blocked by an NCX inhibitor, KB-R7943. This finding further supports a major role of NCX, working in a reverse mode, in the calcium overload during reperfusion following ischemia^22^, and that the inhibition of NCX may decrease calcium overload in ischemia/reperfusion (see^23^).

## Results

### Generation of a transgenic rat strain by combined genetic and phenotypic selection

To establish a rat strain with a single transgene copy per haploid genome, a combined genotype and phenotype screening procedure was applied. First, microinjected zygotes were implanted into pseudopregnant females to be carried to parturition. In order to promote integration and avoid concatemerization of the transgene, the transposon vector was applied as a circular plasmid, together with the SB100X transposase mRNA; the latter one was necessary to minimize mosaicism by providing a translatable source of the transposase during early cell divisions when the host genome is transcriptionally inactive.

**Figure 1:**
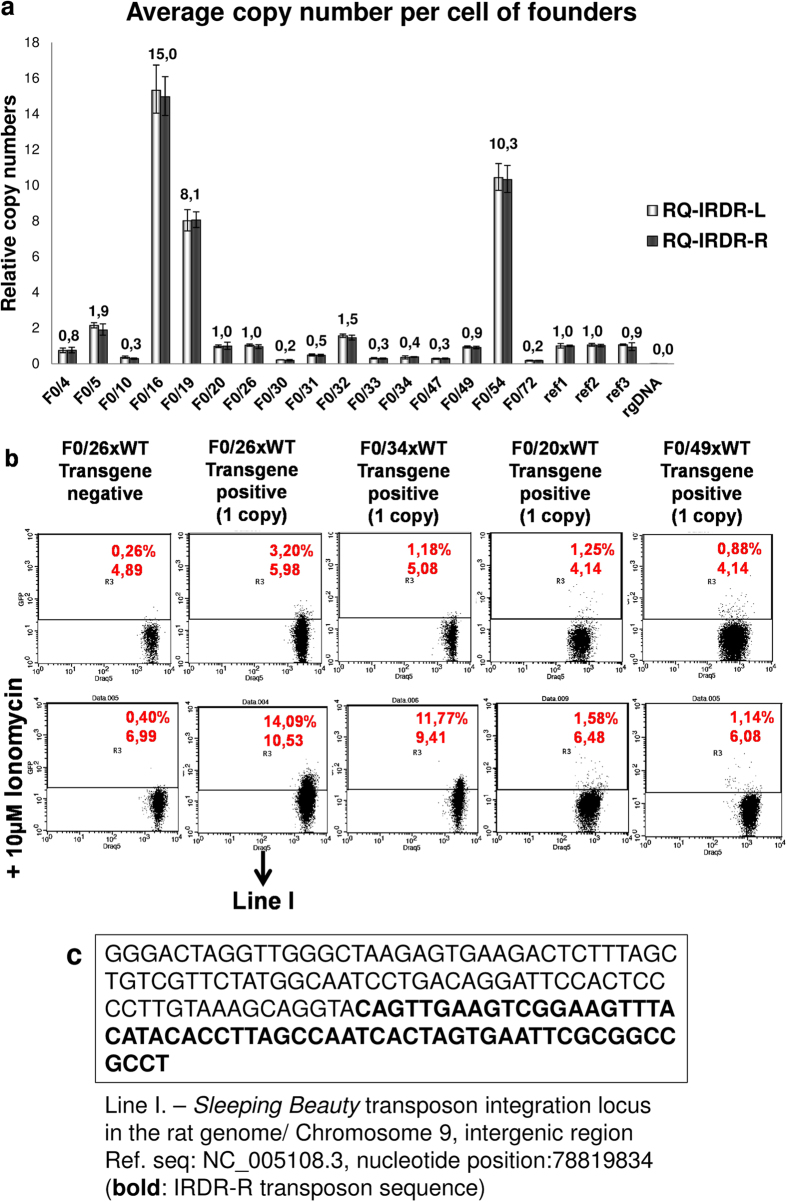
Genotype and phenotype analyses during the establishment of a CAG-GCaMP2 expressing transgenic rat strain. (**A**) Determining the average transgene copy numbers in rat tail tissue samples of the founder (F0) generation using a previously developed, real-time PCR based methodology, applying TaqMan^®^ assays specific for the left and the right inverted repeat-direct repeat transposon sequences (RQ-IRDR-L/R, see Methods). Mean values ± confidence intervals are shown; ref1/2/3 stand for previously genotyped reference clones with one transposon copy, rgDNA is a non-transgenic rat tissue sample (as a negative control). (**B**) Flow cytometry analysis of leukocytes obtained from the blood of transgene positive F1 rats with single-copy integration. A transgene negative F1 rat was used as control. DRAQ5 was used to identify cells with nuclei (leukocytes). 10 μM Ionomycin was used to confirm GCaMP2 expression by testing its functionality. Gated % and median values are indicated by red numbers on the histograms. (**C**) The genomic integration locus of the transgene in the homozygous rat strain as determined by splinkerette PCR.

Next, newborn rats were screened for transgene integration based on their tail tissue-derived gDNA, using SB transposon specific PCR primers. Transposase-induced insertion yielded a high percentage, about 21% of transgenic animals (16 carriers among 75 newborns). To avoid high transgene copy number related phenotypic alterations, low copy number carriers were selected at the F0 generation through determining average transgene copy numbers by a methodology developed earlier^24^. SB copy number distributions were more skewed than expected by earlier studies on cell lines^25^,^26^: apart from 3 extreme cases, the majority of the carriers had copy numbers below 2 (Fig. 1A). The relatively high number of genetically mosaic offspring (copy number <1) was also notable, although two chosen ones showed stable germ line transmission (Table 1).

Founders (F0) with one copy number were crossed with wild type rats to generate low copy number animals with stable transgene integration (F1 generation) (Table 1). Due to its constitutive feature, the CAG promoter driven expression of GCaMP2 was expected to be detectable in leukocytes. In order to accelerate the selection for the desired phenotype, F1 rats with single-copy integration were screened for functional GCaMP2 expression in leukocytes via FACS measurements (Fig. 1B). Single transgene carrier F1 rats with high sensor protein expression were further crossed with wild type counterparts to ensure stable genetic inheritance (F2 generation). GCaMP2 expression levels detected in leukocytes by FACS measurements were later also confirmed to be similar in primary cardiac cells isolated from the same animals (Supplemental Figure 1). Single-copy F2 rats were then inbred to generate homozygous offspring (F3 generation), carrying 2 copy transgenes, one per each haploid genome. Since the F3 generation contains not only homozygous, but also heterozygous and transgene negative animals, our approach provided a special advantage at this point: homozygous rats could be selected by copy number determination, instead of crossing transgenic F3 rats with wild type rats for identification of homozygous ancestor.

Variance in the expression profile of the GCaMP2 protein through subsequent generations and among litter mates was monitored via FACS measurements of ionomycin induced changes in intracellular calcium levels of primary cells isolated from the left ventricular wall (Supplemental Figure 2). The established and constantly maintained homozygous rat strain (‘Line I.’ in Table 1) was proven to be genetically stable: transgene copy numbers and expression profiles were not altered through four subsequent generations. In addition, cell karyotypes were also stable and showed no difference in terms of chromosome numbers and integrity. Transgene integration locus was determined by a splinkerette-PCR method as described earlier^18^: the integration *per se* is not expected to influence gene expression profiles from the neighboring genetic loci (Fig. 1C).

### Expression of the calcium sensor protein in homozygous transgenic rats

We examined if the expression of the calcium sensor caused any phenotypic alterations in transgenic (TG) rats. Brain, lung, spleen, kidney, liver and heart sizes were in all cases similar in the wild-type and homozygous TG rats (*data not shown*). Comparison of heart and body weights did not show any significant differences (heart weight/body weight ratios are (in mg/g): 4.808 ± 0.087 for WT and 4.854 ± 0.356 for TG, n = 8 animals, see Table 2), only a 15% increase was observed in the apical left ventricle (LV) thickness (close to apex, 2.66 mm ± 0.42 for WT and 3.1 mm ± 0.38 for TG, see Table 3), implying a possible LV apical hypertrophy. This result was confirmed by examination of hematoxylin and eosin stained tissue slices prepared from the left ventricle, where only a mild cardiomyocyte hypertrophy was found (Supplemental Figure 3). However, further studies showed no significant pathological alterations in O_2_ saturation (97% ± 0.71 for WT and 95.3% ± 0.47 for TG,), fractional shortening (23.09% ± 6.59 for WT and 29.74 ± 6.54 for TG, obtained by cardiac ultrasound measurements, see Methods) and LV wall thickness at base (close to aorta, 3.38 mm ± 0.6 for WT and 3.57 mm ± 0.33 for TG) in GCaMP2-expressing rats, as compared to wild-type control (Table 3).

**Figure 2:**
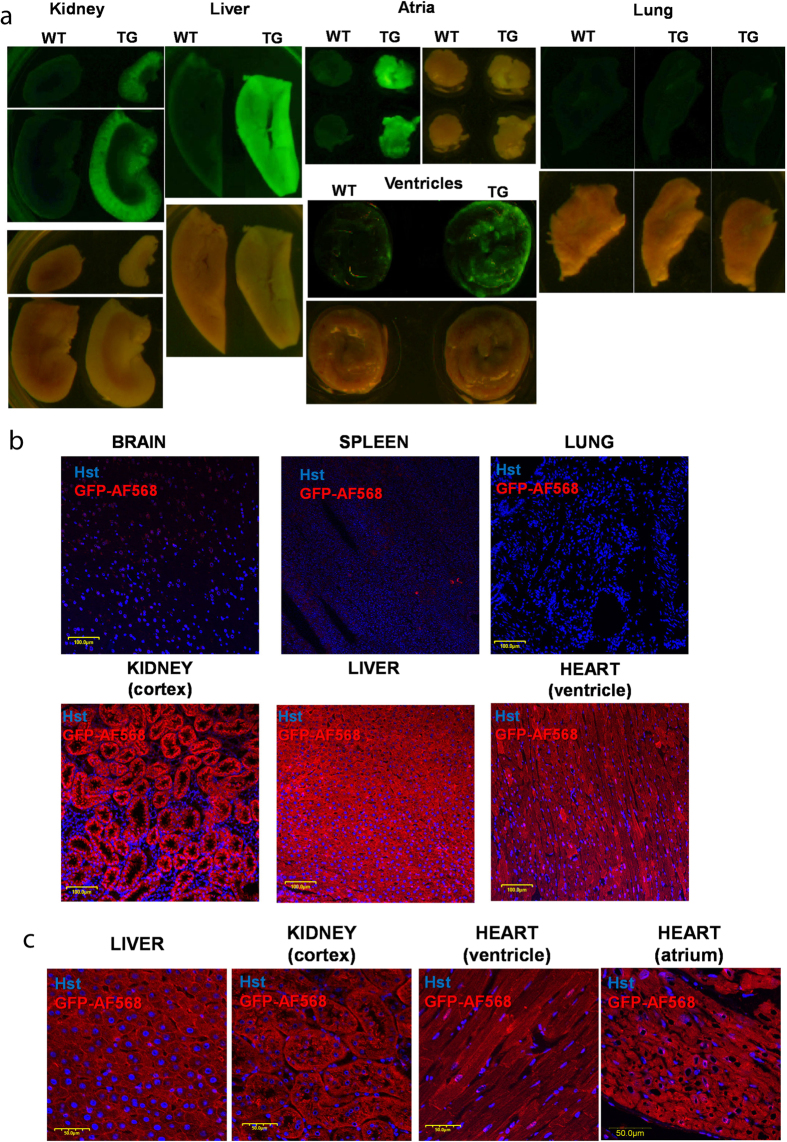
Expression of the GCaMP2 calcium sensor in transgenic rats. (**A**) Fluorescence (upper) and bright field (lower) images of wild type (WT) and transgenic (TG) kidney, liver, heart atria, ventricles and lung. (**B**) Immunohistochemistry of transgenic brain, spleen, lung, renal cortex, liver, and heart ventricle at lower magnification (scale bars represent 100 μm). (**C**) Immunohistochemistry of transgenic liver, renal cortex, heart ventricle and atrium at higher magnification (scale bars represent 50 μm). Immunostaining of the GCaMP2 was performed by a GFP-recognizing antibody. Hoechst (Hst) was used to stain the nuclei.

Fluorescence images of brain, lung, spleen, kidney, liver, atria and ventricles of TG and WT rats were compared using an *in vivo* imaging system to confirm GCaMP2 expression. Kidney, liver and heart, especially the atria were found to have high GCaMP2 fluorescence intensities (Fig. 2A), while GCaMP2 expression in the lung, brain and spleen failed to reach detectable levels (*data are shown only for lung*). The observed differences among the different tissues could have been caused either by different intracellular calcium levels or different GCaMP2 expression levels. Therefore, we examined GCaMP2 protein expression in samples obtained from different organs of TG rats by immunohistochemistry, and confirmed higher protein levels of GCaMP2 in the kidney, liver and heart samples while in brain, lung and spleen measurable GCaMP2 expression could not be detected (Fig. 2B,C). Functional expression of GCaMP2 could be confirmed in isolated primary cardiac (Supplemental Figure 2), renal^27^ and hepatic cells (*unpublished observations by L. Homolya*). Consistent with higher protein levels of GCaMP2, higher mRNA expression could be confirmed in the kidney, liver and heart samples, while brain, lung and spleen were found to express low levels of the transgene mRNA (Supplemental Figure 4A). Low variance in mRNA expression levels among homozygous GCaMP2 rats was confirmed when two rats from the same litter were analyzed (Supplemental Figure 4A). This finding was in good agreement with the low variance of GCaMP2 expression measured by FACS in cardiac primary cells isolated from littermates (Supplemental Figure 2). Differences in protein expression intensities in the kidney between cortex and medulla or in the heart between atrium and ventricles detected by the fluorescent *in vivo* imaging system were also present at the transcriptional level (Supplemental Figure 4B and C).

### Direct examination of calcium signaling in cardiomyocytes by using CAG-GCaMP2 transgenic rats

**Figure 3:**
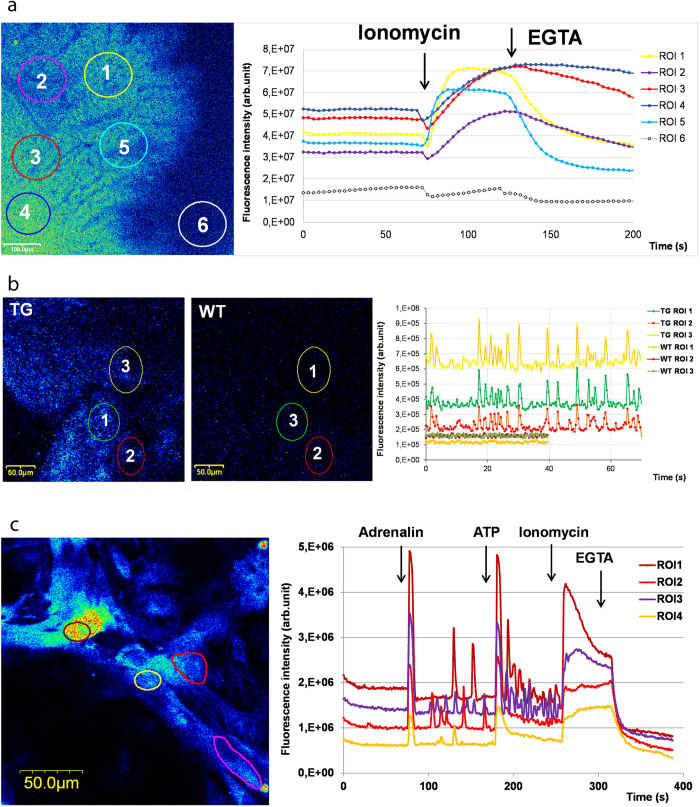
CAG-GCaMP2 expressing ventricular slices and cardiac cultures allow detection of spontaneous and ligand-induced calcium signals. (**A**) The left panel shows the fluorescence image of a ventricular slice obtained from a transgenic rat heart with circled region of interest (ROI). The right panel shows the effect of ionomycin and EGTA administration on the kinetics of calcium signals in the indicated ROIs. (**B**) The left panel shows the fluorescence images of transgenic (TG) and wild type (WT) ventricular slices with circled region of interest (ROI). The right panel shows the fluorescence signals caused by spontaneous calcium waves in TG and WT ventricular slices in the indicated ROIs. (**C**) Spontaneous calcium oscillations were induced by adrenalin and ATP in ventricular cell cultures generated from GCaMP2 expressing rats. Cell cultures obtained from the heart of TG rats were treated with adrenalin, ATP, ionomycin and EGTA. Physiologically relevant ligands such as adrenalin or ATP evoked a strong increase in intracellular Ca^2+^ levels and both compounds induced spontaneous calcium oscillations after addition.

Acute ventricular slices were characterized for ionomycin-induced Ca^2+^ signals to confirm the functional expression of the GCaMP2 protein (Fig. 3A). In the TG tissue slices, intracellular Ca^2+^ overload could be evoked by 10 μM ionomycin, initiating a wave of calcium rise running through the slices, while intracellular calcium could be depleted by 10 mM EGTA (Supplemental movie 1). Ionomycin and EGTA administration also induced small changes in autofluorescence in the WT animal heart slices, however, these changes were two orders of magnitude smaller than the GCaMP2-dependent signals in the TG slices.

Freshly prepared ventricular slices often showed spontaneous contractions, resulting in fluorescence intensity changes in TG slices (Fig. 3B). Spontaneous contractions were localized within the tissue slices and did not generate major artifacts, as confirmed by the absence of changes in the autofluorescence signal in spontaneously contracting WT ventricular slices (Fig. 3B). Thus acute tissue slices can be properly used to follow the calcium transients and contractions in the TG heart tissue samples.

The TG CAG-GCaMP2 rat provides a stable and constant source of primary cardiac cells, suitable for a detailed examination of calcium signals in imaging-based drug screening applications. Therefore we examined the functionality of the GCaMP2 calcium indicator protein in primary cells isolated from the atria or from the ventricles. Cardiomyocyte-identity of the isolated cells was confirmed by immunostaining against the cardiac Troponin T and the cardiac isoform of the NCX protein (Supplemental Figure 5).

**Figure 4:**
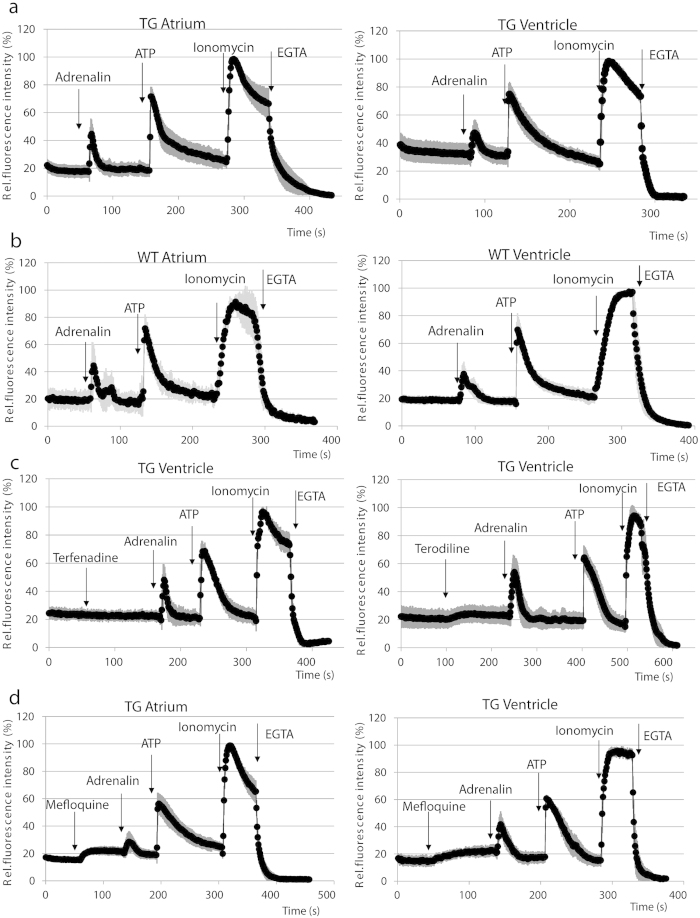
CAG-GCaMP2 expressing cardiomyocyte cultures allow detection of ligand-induced calcium signals and drug-induced alteration of physiologically relevant ligand-induced calcium signals. (**A**) The effects of adrenalin, ATP, ionomycin and EGTA administration are shown on the kinetics of calcium signals in atrial (left panel) and in ventricular (right panel) cardiomyocyte cultures. (**B**) Ligand-induced calcium signals in wild type (WT) primary cardiac cultures loaded with the calcium-sensitive Fluo-4 AM dye. (**C**) The effects of 6.0 μM terfenadine (left panel) or 10.0 μM terodiline (right panel) followed by adrenalin, ATP, ionomycin and EGTA administration are shown on the kinetics of calcium signals in ventricular cell cultures. (**D**) The effects of 37.5 μM mefloquine, as well as adrenalin, ATP, ionomycin and EGTA administration are shown on the kinetics of calcium signals in atrial (left panel) and in ventricular (right panel) cell cultures. For each measurement, a total number of at least 25 cells was examined; light gray lines represent S.E.M. values.

In order to document the applicability and limitations of this system, isolated and cultured rat ventricular cardiomyocytes were characterized for spontaneous and ligand-induced Ca^2+^ signals by confocal microscopy. At high magnification, administration of 10 μg/ml adrenalin caused a well measurable oscillation, while 100 μM ATP further increased the frequency of oscillations in ventricular cells without reaching the limit of detection (Fig. 3C). Detection of calcium signals at lower magnification provided a more high throughput application by calculating the average of Ca^2+^ transients evoked by adrenalin and ATP in atrial and ventricular cultures, while oscillations of single cells was still detectable as shown by the high S.E.M. values (Fig. 4A). For validation of calcium signals evoked by adrenalin and ATP, similar measurements were carried out with Fluo4-AM loaded cardiomyocytes isolated from WT animals (Fig. 4B), resulting in similar calcium signals.

To document the applicability of this system for drug testing, we have examined the effect of 6 μM terfenadine and 10 μM terodiline on the basal calcium levels in ventricular myocytes. We used terfenadine instead of its active metabolite fexofenadine (terfenadine carboxylate), a potent antihistamine agent because it was reported that terfenadine itself is the likely cause of the cardiotoxic effect^8^,^28^. Treatment with 10 μM terodiline caused elevation in basal calcium levels, moreover, terodiline pre-treatment resulted in a significant increase in the amplitude of adrenalin-induced calcium transients (Fig. 4C). The ATP-evoked calcium influx was also elevated when ventricular cultures were pre-treated with terodiline or terfenadine, while terfenadine had only moderate effect on the adrenalin-induced calcium influx (Supplemental Figure 6).

We used the GCaMP2 rat system to examine the effect of an antimalarial, and potentially cardiotoxic agent, mefloquine, on the calcium signal responses in the primary cardiomyocyte cultures. As shown (Fig. 4D), higher mefloquine concentrations induced an increase in cellular calcium levels both in the atrial and ventricular CMs, but mefloquine had different effects on the ligand-induced signals. Atrial CMs showed a decrease in ligand-induced Ca^2+^ influx, however, the upstroke kinetics of the calcium transients were not significantly altered (Supplemental Figure 7A and B). Ligand-induced calcium signals in ventricular CMs were affected in their upstroke kinetics by mefloquine, as shown in Supplemental Figure 7A and B, that is mefloquine-treated ventricular CMs showed higher ‘time to peak’ values as compared to control ventricular CMs. It also appeared that adrenalin induced calcium signals were more affected by mefloquine than the ATP-induced signals.

We found that a 24 hours pre-treatment with near therapeutic (2.6 μM) concentrations of mefloquine did not increase the basal calcium levels in cardiomyocytes, however, calcium signals induced by adrenalin or ATP were similarly altered as after the immediate addition of 37.5 μM mefloquine (Supplemental Figure 7A).

**Figure 5:**
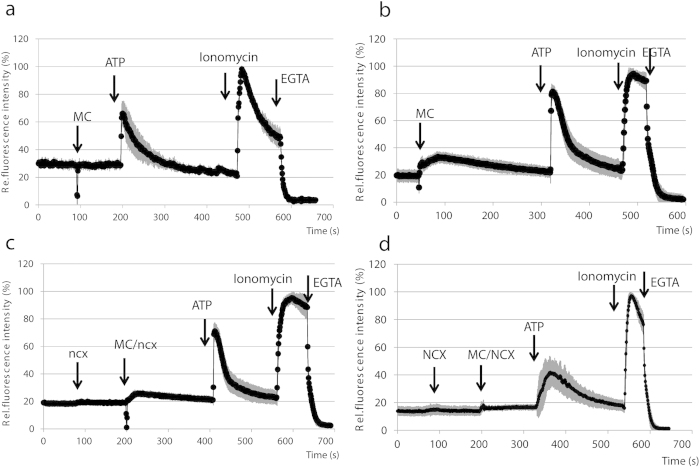
Modeling hypoxia/reoxygenation in CAG-GCaMP2 expressing ventricular cultures allows detection of the protecting effect of KB-R7943. (**A**) Control measurement in ventricular cultures without CoCl_2_ pre-treatment, showing no changes in fluorescence by medium change (MC). (**B**) In CoCl_2_-pretreated ventricular cultures, reoxygenation through medium change (MC) causes a significant calcium increase in hypoxic ventricular cells. (**C**) In CoCl_2_-pretreated ventricular cultures, administration of 0.5 μM KB-R7943 (ncx) reduced the calcium influx caused by reoxygenation. (**D**) In CoCl_2_-pretreated ventricular cultures, administration of 1 μM KB-R7943 (NCX) eliminates the calcium influx caused by reoxygenation.

After the occurrence of a cardiac hypoxia, (e.g.: by acute myocardial infarction), reperfusion causes major cell death in patients mostly through an altered calcium handling^29^. To mimic ischemia (hypoxia) and reperfusion (reoxygenation) at an *in vitro* cellular level, we pre-treated rat primary ventricular cells with 400 μM CoCl_2_, and after 24 hours we rapidly exchanged the medium for an oxygenated one, containing no CoCl_2_. Reoxygenation caused a significant calcium increase, while the medium change without CoCl_2_ pre-treatment did not alter the intracellular calcium levels (Fig. 5A,B).

At the time of reperfusion/reoxygenation, following the ATP depletion and intracellular Na^+^ increase produced by hypoxia, the reverse-mode activation of the NCX has been suggested to be a key factor in cellular calcium loading^22^. Thus the inhibition of NCX and the related calcium influx during initial reperfusion may reduce myocardial injury^23^. In order to explore this effect, different concentrations of the NCX inhibitor, KB-R7943, were added to the primary ventricular cells at the time of reoxygenation. This treatment, in a concentration dependent manner, decreased or eliminated the rise in cellular calcium due to reoxygenation (Fig. 5C,D), although the amplitude of ATP-driven Ca^2+^ influx was also lowered at the higher concentration.

## Discussion

In the present study we describe the generation and validation of a new GCaMP2 expressing rat strain with a single transgene copy per haploid genome. This transgene model was generated by a *Sleeping Beauty* transposon-based method, and the transgene expression is driven by a CAG promoter variant^18^. Utilizing the constitutive nature of this promoter, a combined genotype/phenotype screening approach was applied to establish a homozygous rat strain in a relatively short period of time. In accordance with our earlier findings with embryonic stem cells^30^, the highest GCaMP2 expression was found in the cardiac tissues, especially in the atria, while appreciable transgene expression was found in the kidney and in the liver. Anatomical analyses found no differences in the heart size and weight between WT and TG animals, while slight left ventricular wall thickening was observed at the apex of the heart in transgenic rats. However, this did not cause any alterations in the heart function and other circulatory parameters, since physiological parameters of the CAG-GCaMP2 expressing rats were similar to the WT rats.

As opposed to the traditional detection methods of filling up the cells with a calcium-sensitive, often toxic fluorescent dyes, the genetically encoded and stably expressed calcium indicators have a clear advantage of providing a non-invasive platform for calcium measurements. Although new generation calcium sensors (e.g. GCaMP3–8) are already available^31^,^32^,^33^,^34^, most of them have higher rates of induced cytomorbidity which limits their applications^35^. In addition, they were developed to follow action potentials of neurons more reliably, while GCaMP2 offers an effective system of Ca^2+^ signal measurements in all other tissues with potentially less toxicity and side effects. The physiological relevance of the calcium sensor expressing rat model was verified by the characterization of ligand-induced Ca^2+^ signals both *ex vivo* and *in vitro*. In acute ventricular slices, when spontaneous contractions were observed, the GCaMP2 fluorescence intensity increased parallel with the contractions. Experiments in primary cell cultures isolated from TG rats were performed using both atrial and ventricular cells. As documented in Supplemental 5, these cultured cells were mainly troponin T (cTnT) positive cardiomyocytes, and also showed high expression of the calcium sensor protein GCaMP2.

In this report we have examined the effect of an antimalarial agent, mefloquine, on the calcium signal responses in isolated cardiomyocytes. Mefloquine, a widely used agent to prevent and treat *Plasmodium falciparum* malaria, has a suspected cardiotoxicity. Mefloquine alone did not cause significant alterations in a healthy heart muscle^36^, while this compound induced changes in the heart calcium levels through the modulation of the L-type Ca^2+^ current^19^. In addition, pre-treatment with mefloquine potentiated the effects of halofantrine on the prolongation of QT intervals in anaesthetized rabbits^37^.

These studies raised warnings against the use of mefloquine in the case of cardiac problems. Our present experiments reported here indicate that in the rat cardiomyocyte model a long (24 hours) pre-treatment with low (near therapeutic) concentrations of mefloquine did not increase the basal calcium level, but considerably decreased the calcium signals induced by adrenaline or ATP, especially in the atrial cells (Supplemental Figure 7A and B). Higher mefloquine concentrations induced a significant increase in intracellular calcium levels (Fig. 4D). Moreover, mefloquine treatment also altered the properties of Ca^2+^ transients both in atrial and in ventricular cells (Supplemental Figure 7A and B). These findings raise the possibility for a direct effect of mefloquine on cardiomyocyte calcium homeostasis. In addition, two drugs, terodiline and terfenadine withdrawn from the market due to QT interval prolonging effect, were used to validate the CAG-GCaMP2 rat system as a potent tool for predicting cardiotoxic side effects. Both terodiline^38^ and terfenadine inhibits the rapid component of the delayed-rectifier K^+^ current, therefore the increase in calcium influx detected may be a consequence of the reduction of active K^+^ channels, normally compensating calcium influx with K^+^ efflux in ventricular cardiomyocytes.

Our results, obtained in an *in vitro* hypoxia-reoxygenation model, were in agreement with previous studies showing that cellular Ca^2+^ levels increase at the time of reoxygenation (Fig. 5B)^22^,^29^, and this elevation can be eliminated by the NCX inhibitor KB-R7943 (Fig. 5D)^23^. Moreover, we found a reduced calcium signal induced by ATP after the addition of 1 μM KB-R7943, showing the potential role of NCX in the calcium signaling of ventricular cells.

This study is the first publication dealing with the generation of a TG rat strain, stably expressing a calcium sensor fluorescent protein. While mouse models expressing genetically engineered calcium indicators have already been described^14^,^39^ the present rat model with calcium sensor expression may significantly improve the experimental background examining these phenomena in a widely used system for pharmacological and toxicological studies. The transgenic rats expressing a calcium-sensitive protein combined with recently developed, genetically engineered disease-specific rat strains (see SAGE, KO Rat Consortium, http://www.sageresearchlabs.com/research-models), could provide a valuable system for further elucidation of the mechanisms and potential treatments of heart diseases.

## Methods

All animal protocols were approved by the Hungarian Animal Health and Animal Welfare Directorate according to the European Union’s most recent directives. All surgical procedures were performed according to the Committee on the Care and Use of Laboratory Animals of the Council on Animal Care at the National Institute of Oncology in Budapest, Hungary (22.1/722/3/2010).

### Rat transgenesis

To establish a rat strain with a single transgene copy per haploid genome (two copies per animal), a combined genotype and phenotype screening procedure was applied (see Results). Transgenic rats were derived by microinjection of zygotes from superovulated donor females of an outbred Sprague-Dawley strain, and transgenic embryos were implanted into pseudopregnant Wistar female rat recipients (experiment license number in Hungary: PEI/001/2197-2/2013). Circular transposon plasmid vector^15^ was applied together with the SB100X transposase mRNA. The latter translation competent mRNA was prepared by *in vitro* transcription using the mMessage mMachine^®^ T7 kit (Life Technologies). For genotyping, genomic DNA (gDNA) was extracted from rat tails as described earlier^17^, and the following PCR primers were used to detect the transposon: 5′- AATTCCCTGTCTTAGGTCAGTTAGGA and 5′- TTCAGGTTCAGGGGGAGGTGTGGG. As a rat gDNA control, primers 5′- AAGATTGAATGTCTGTAAGTTCGAG and 5′- TGTAATTGGTTTGGGGTTAT were used to amplify a sequence from intron 1 of the RN-LOC500546 gene. SB transposon copy numbers were determined as described earlier^24^.

### Organ processing

Animals were sacrificed by overdosing a 4-component anesthetic mixture (20 mg/kg zolazepam, 12.5 mg/kg xylazine, 3 mg/kg butorphanol, 20 mg/kg tiletamine). The organs were removed, washed twice in PBS and were either archived for later use by snap freezing with Isopentane or fluorescent images were made by placing the organs into an *in vivo* imaging system (LT-9MACIMSYSPLUSC, Lightools Research). For experiments with ventricular slices the left ventricle was cut into 300 μm thin section with a McIlwain tissue chopper (Mickle Laboratory Engineering Comp.).

### Structural and functional characterization of cardiac muscle in transgenic and wild type animals

Fractional shortening, basal and apical thickness of the left ventricle (LV) and oxygen saturation were measured in both transgenic and wild type rats. The end-diastolic and end-systolic dimensions (EDD and ESD) of the LV were measured in M-mode by a GE LOGIQ 5 Pro ultrasound system (General Electric Healthcare) under 4% isoflurane anaesthesia, which was introduced directly without previous prenarcotic pretreatment, and fractional shortening (FS) was calculated using the following equation:


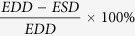


All ultrasound examinations were performed in a 5 minutes interval after anaesthesia to avoid possible pathological alterations due to anesthetic inhalation, and O_2_ saturation was monitored in the hind paw with a pulse oximeter (Pulse-BLT M700 Vet). After echocardiography the animals were euthanized with a single intracardial injection of barbiturate (Euthasol® 40% inj. A.U.V., Produlab Pharma b.v.), the heart was removed, and the basal (aprox. 3 mm from the aortic valve) and apical (3 mm from apex of the heart) LV wall thickness was measured by a caliper at least twice. The hearts were fixed in formalin, embedded in paraffin and haematoxylin-eosin stained for micro-structural analysis.

### Real-time PCR analysis

Frozen rat samples were pulverized under liquid nitrogen and were homogenized in TRIzol™ Reagent (Life Technologies). Total RNA was isolated from tissue samples and RNA degradation was monitored by gel electrophoresis. cDNA samples were prepared from 1 μg total RNA using the Promega Reverse Transcription System Kit. The Pre-Developed TaqMan® assay for beta-2-microglobulin (B2M) (Life Technologies) was used as endogenous control in real-time qPCR experiments; for quantifying EFGP mRNA, specific TaqMan® assay was designed for the cDNA^18^. Real time PCR analyses were carried out using the StepOne^TM^ Real-Time PCR System (Life Technologies); mRNA fold changes were determined using the 2^−ΔΔCt^ method. Relative mRNA levels were presented as mean values ± S.E.M. of 3 independent experiments.

### Generation of primary cell cultures from cardiac atria and ventricles

Primary cell cultures were established from the atria or the ventricles of 10–30 days old male TG and wild type rats. Briefly, the left and right atria were separated from the ventricles and were cut into ~1 mm^3^ pieces, similarly to the walls of the right and left ventricles. The pieces of the atria or the ventricles were transferred to a 50 ml conical tube containing 20 ml enzyme mixture of 200 U/ml type IV collagenase and 0.6 U/ml dispase (Life Technologies), vortexed for 1 minute then digested for 15 minutes at 37 °C. After 15 minutes the larger pieces were settled, the supernatant was transferred to a sterile conical tube, centrifuged at 300 × g for 5 minutes and the pellet was dissolved in pure fetal bovine serum (FBS). The remaining tissue pieces were re-digested with fresh enzyme mix. This procedure was repeated until no macroscopic pieces were observed. The cell suspension was filtered through a 70 micron nylon filter insert (BD Biosciences), centrifuged for 5 minutes at 300 × g, the supernatant was removed and the pellet was dissolved in complete DMEM (2% penicillin/streptomycin + 1% glutamine + 10% FBS, 25 mM HEPES). Cells in 300  μL of complete DMEM were plated onto 8-well Nunc Lab-Tek II Chambered Cover glass (Nalgen Nunc International) covered with 0.1% gelatin. After 90 minutes the supernatant from all chambers were transferred to a clean, gelatin coated chamber to enrich cardiomyocytes and decrease the number of fibroblasts. For experiments only secondary chambers were used and culture media were changed on every second day.

### Immunohistochemistry

Snap-frozen tissues were cut with a cryostat into 5 μm thin sections. The tissue slices were transferred onto microscope slides and were incubated with 10 mM EGTA for 5 minutes before fixation with methanol. Tissue sections were then washed in PBS, blocked with 2% bovine serum in PBS (1 hr) and incubated with a rabbit polyclonal antibody (1:500) targeting GFP (Abcam, ab290) and with a mouse monoclonal antibody (1:100) against cardiac Troponin T (Abcam, ab8295) in 0.5% bovine serum in PBS overnight at 4 °C. After a 90 minutes of PBS wash, tissue slices were incubated (1 hr) with Alexa Fluor 488 or Alexa Fluor 568-conjugated secondary antibody against rabbit IgG and with Alexa Fluor 568-conjugated secondary antibody against mouse IgG (all by 1:200; Life Technologies), washed in PBS and the nuclei were stained with Hoechst 33342 dye. Stained sections were mounted with ProLong Gold (Life Technologies). Wild type tissues were used as negative controls. Immunohistochemistry images were examined by an Olympus FV500-IX confocal laser scanning microscope.

### Immunocytochemistry

Seven day old cultures of primary cells isolated from rat atrium or ventricle were incubated with 10 mM EGTA for 5 minutes before methanol fixation and washed with PBS. Blocking and immunostaining were performed as described above, nuclei were stained with DAPI. For staining of the Na^+^/Ca^2+^ exchanger (NCX) the R3F1 antibody was used, kindly provided by Michela Ottolia from the Cedars-Sinai Heart Institute. Isotype and secondary antibody controls were used, and image detection was performed as described above.

### Flow cytometry measurements (FACS)

Leukocytes were obtained through red blood cell lysis, while primary ventricular cells were obtained as described earlier. The isolation of primary rat hepatocytes were performed by a three-step collagenase perfusion method^40^. Renal cells were isolated from the cortex after decapsulation and separation from the renal medulla, followed by enzymatic digestion steps as described previously^27^. Measurements were carried out in HBSS (Life Technologies). In the case of blood samples DRAQ5 was used to identify cells with nuclei (leukocytes) and 10 μM Ionomycin was used to confirm GCaMP2 expression by testing its functionality. For primary ventricular, renal cortical cells, and hepatocytes 5 μM Ionomycin was used to confirm GCaMP2 functionality. Forward scattered light (FSC channel), GCaMP2 expression (FL1 channel) and DRAQ5 staining (FL4 channel) were detected by a BD FACSCalibur flow cytometer.

### Calcium signal measurements

GCaMP2 expressing primary cardiomyocytes were seeded onto eight-well Nunc Lab-Tek II Chambered Coverglass (Nalge Nunc International) covered by 0.1% gelatine. Culture media was removed by changing the medium to HBSS. In the case of *ex vivo* measurements 300 μm thin ventricular sections were placed into HBSS containing MatTek glass-bottom dishes. Ligand concentrations were chosen according to literature: ATP (100 μM), adrenalin (10 μg/ml) or ionomycin (5 μM for *in vitro* cultures and 10 μM for tissue slices). For calibration, EGTA was used in 5x excess of calcium concentration in the medium^41^. Calcium signal measurements were carried out by following time lapse sequences of cellular fluorescence recordings, and images were analyzed with the FluoView Tiempo (v4.3, Olympus) software. For GCaMP2 imaging, the 488 nm laser line was used for excitation and emission was measured between 505 and 535 nm. In confocal images artificial coloring was used for better visualization. Fluo-4 imaging was carried out as described previously^41^.

### Induction of hypoxia-reoxygenation in primary cells

To mimic hypoxia in cultured cardiomyocytes, 400 μM of cobalt chloride (CoCl_2_) was added in 500 μL phenol-free completed media and incubated in normoxia (21% O_2_). 24 hours later the cells were transferred to the confocal microscope stage and the baseline cellular calcium levels were measured. Thereafter the culture medium was rapidly changed to phenol-free HBSS without CoCl_2_, and changes in fluorescent intensities were detected continuously.

## Additional Information

**How to cite this article**: Szebényi, K. *et al.* Generation of a Homozygous Transgenic Rat Strain Stably Expressing a Calcium Sensor Protein for Direct Examination of Calcium Signaling. *Sci. Rep.* **5**, 12645; doi: 10.1038/srep12645 (2015).

## Supplementary Material

Supplementary Figures

Supplementary Movie 1

## Figures and Tables

**Table 1:** Genotype analyses of the F1 generation offspring of four chosen F0 individuals crossed with wild type rats.

	Wild type crossings of founders							
	(Line II.)		(Line I.)		(Line III.)		(Line IV.)	
Parents	♂	♀	♂	♀	♂	♀	♂	♀
	F0/20	WT	F0/26	WT	F0/34	WT	F0/49	WT
Average copy	**1.0**	**–**	**1.0**	**–**	**0.4**	**–**	**0.9**	**–**
F1	Progeny	Copy	Progeny	Copy	Progeny	Copy	Progeny	Copy
	F1/36	0	F1/68	1	F1/51	0	F1/17	1
	F1/37	1	F1/69	0	F1/52	0	F1/18	0
	F1/38	1	F1/70	1	F1/53	1	F1/19	0
	F1/39	0	F1/71	1	F1/54	0	F1/20	0
	F1/40	0	F1/72	0	F1/55	1	F1/21	1
	F1/41	0	F1/73	0	F1/56	0	F1/22	0
	F1/42	0	F1/74	1	F1/57	0	F1/23	0
	F1/43	2	F1/75	1	F1/58	1	F1/24	1
	F1/44	1	F1/76	0	F1/59	0	F1/25	1
	F1/45	0	F1/77	1	F1/60	0	F1/26	1
	F1/46	0	F1/78	1	F1/61	1	F1/27	0
	F1/47	0	F1/79	0	F1/62	0	F1/28	1
	F1/48	0	F1/80	0	F1/63	1	F1/29	0
	F1/49	0	F1/81	1	F1/64	0	F1/30	0
	F1/50	1	F1/82	0	F1/65	0	F1/31	0
			F1/83	1	F1/66	0	F1/32	1
			F1/84	0	F1/67	1	F1/33	1
			F1/85	0			F1/34	0
							F1/35	1

Based on further genetic and phenotypic characterizations, ‘Line I.’ rat line was used to establish the homozygous rat strain with one copy CAG-GCaMP2 transgene per haploid genome. Of note that although the F0/34 founder of ‘Line III.’ was a genetic mosaic (average copy number below 1), it stably inherited one transgene copy in its germline (see F1 animals).

**Table 2:** Body and heart weight of wild type (WT) and transgenic (TG) rats. The data represents mean values ± S.E.M (n=8).

*n*	WT	TG
	8	8
body weight (g)	198 ± 29.399	213.375 ± 48.382
heart weight (mg)	953.625 ± 152.669	1032.750 ± 226.265
heart weight/body weight ratio (mg/g)	4.808 ± 0.087	4.854 ± 0.356

**Table 3:** Comparison of body and heart parameters of wild type (WT) and CAG-GCaMP2 (TG) rats.

	Weight (g, SD)	O2 saturation (%, SD)	Fractional shortening (%, SD)	LV Thickness at base (mm, SD)	LV Thickness at apex (mm, SD)
**WT (n = 4)**	203.75 ± 14.74	97 ± 0.71	23.09 ± 6.59	3.38 ± 0.6	2.66 ± 0.42
**TG (n = 3)**	240.67 ± 22.9	95.33 ± 0.47	29.74 ± 6.54	3.57 ± 0.33	3.1 ± 0.38
