# Supplementary material for: Generation of a Homozygous Transgenic Rat Strain Stably Expressing a Calcium Sensor Protein for Direct Examination of Calcium Signaling
Source: Sci Rep. 2015 Aug 3;5:12645. doi: 10.1038/srep12645 (PMC4522653; doi:10.1038/srep12645)
Supplement: Supplementary Figures [file srep12645-s1.doc]

**Generation of a Homozygous Transgenic Rat Strain Stably Expressing a Calcium Sensor Protein for Direct Examination of Calcium Signaling**

Kornélia Szebényi^1^, András Füredi^1^, Orsolya Kolacsek^1^, Enikő Pergel^1^,

Zsuzsanna Bősze^2^, Balázs Bender^3^, Péter Vajdovich^4^, József Tóvári^5^,

László Homolya^1^, Gergely Szakács^1^, László Héja^6^, Ágnes Enyedi^7^,

Balázs Sarkadi^1,8^, Ágota Apáti^1^ and Tamás I. Orbán^1^

^1^ Institute of Enzymology, Research Centre for Natural Sciences, Hungarian Academy of Sciences, Budapest, Hungary

^2^ NARIC-ABC, Gödöllő, Hungary

^3^ ImmunoGenes Ltd., Budakeszi, Hungary

^4^ Department of Clinical Pathology and Oncology, Faculty of Veterinary Science, Szent István University, Budapest, Hungary

^5^ Department of Experimental Pharmacology, National Institute of Oncology, Budapest, Hungary

^6^ Institute of Organic Chemistry, Research Centre for Natural Sciences, Hungarian Academy of Sciences, Budapest, Hungary

^7^ 2^nd^ Institute of Pathology, Semmelweis University, Budapest, Hungary

^8^ Department of Biophysics and Radiation Biology, Semmelweis University, Budapest, Hungary and MTA-SE Molecular Biophysics Research Group, Budapest, Hungary

**Supplemental Figures and Figure Legends**

***
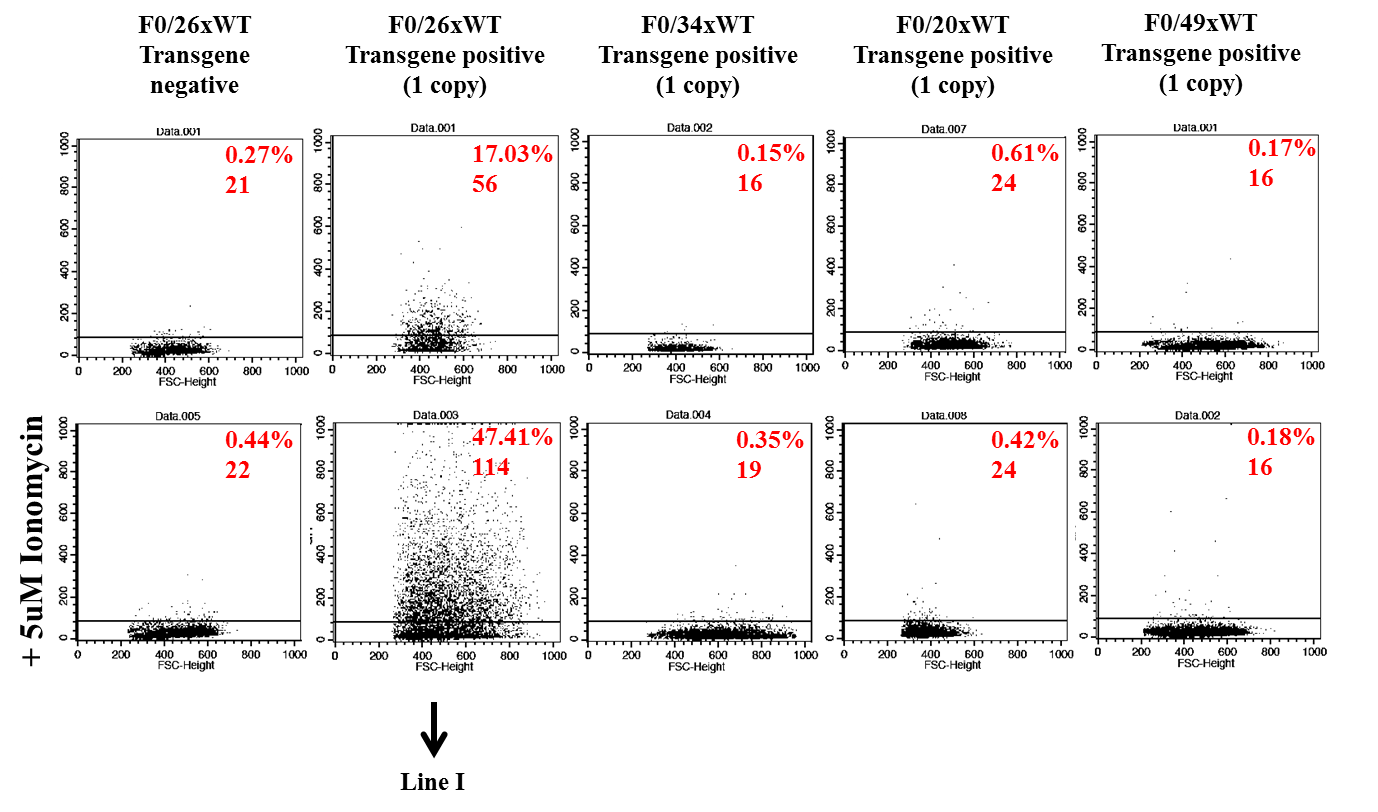
***

***Supplemental Figure 1***.

**Flow cytometry analysis of primary cells isolated from left ventricular wall of single transgene carrier F1 rats.** Primary ventricular cells obtained from a transgene negative rat were used as control. 5 µM Ionomycin was used to assess functional expression of the GCaMP2 protein. Gated % and median values are indicated by red numbers on the dot plots. Forward scattered light was detected in FSC, GCaMP2 expression was detected in FL1 (GFP) channel.


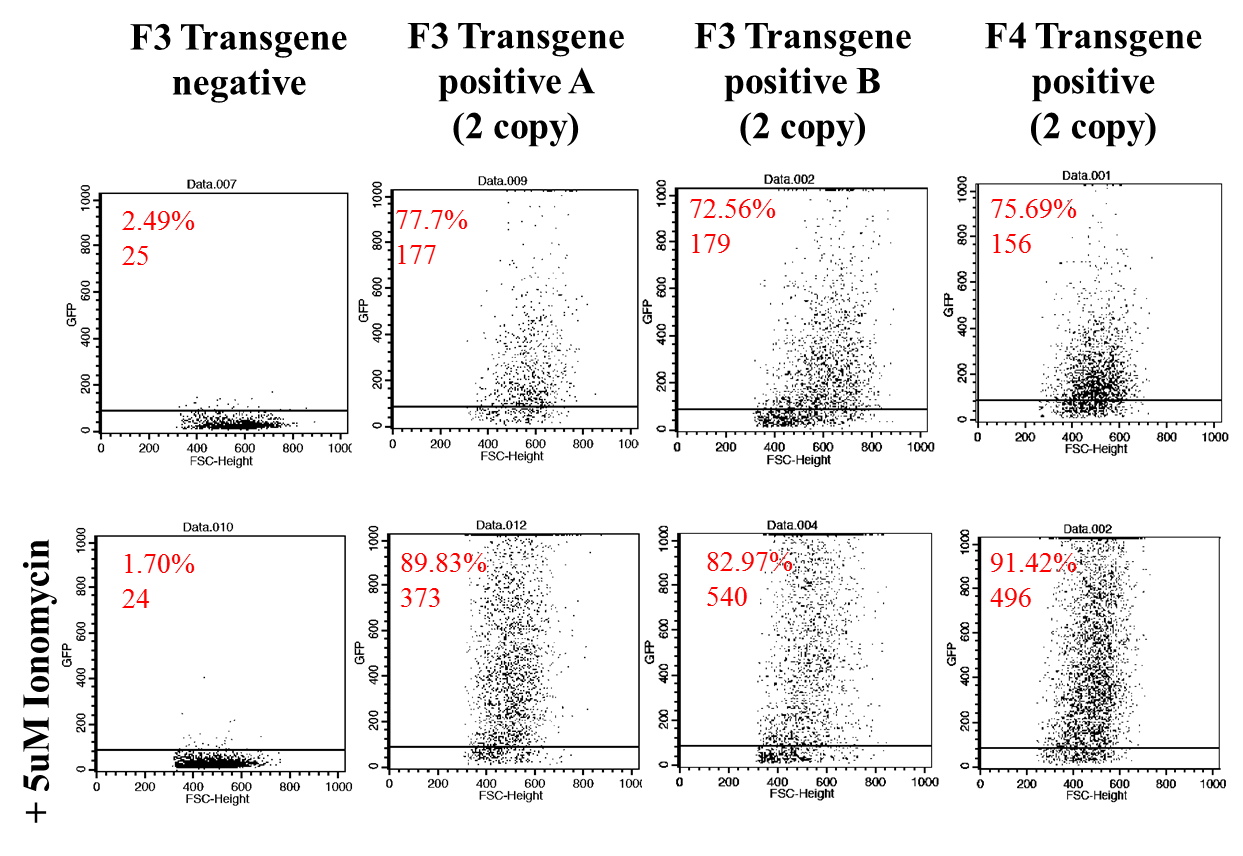


***Supplemental Figure 2***.

**Flow cytometry analysis of primary cells isolated from left ventricular wall of homozygous F3 and F4 transgenic rats.** Samples indicated by A and B were obtained from two different animals born in the same litter. Primary ventricular cells obtained from a transgene negative rat were used as control. 5 µM Ionomycin was used to assess functional expression of the GCaMP2 protein. Gated % and median values are indicated by red numbers on the histograms. Forward scattered light was detected in FSC, GCaMP2 expression was detected in FL1 (GFP) channel.

***Supplemental Figure 3***.

**Analysis of the heart of CAG-GCaMP2 expressing homozygous (F4) and wild type (WT) rats.** Wild type (WT) and transgenic (TG) hearts, showing no significant differences in shape or size. Hematoxylin (blue for nuclei) and eosin (red for cytoplasm) staining of paraformaldehyde-fixed and paraffin-embedded left ventricular slices.


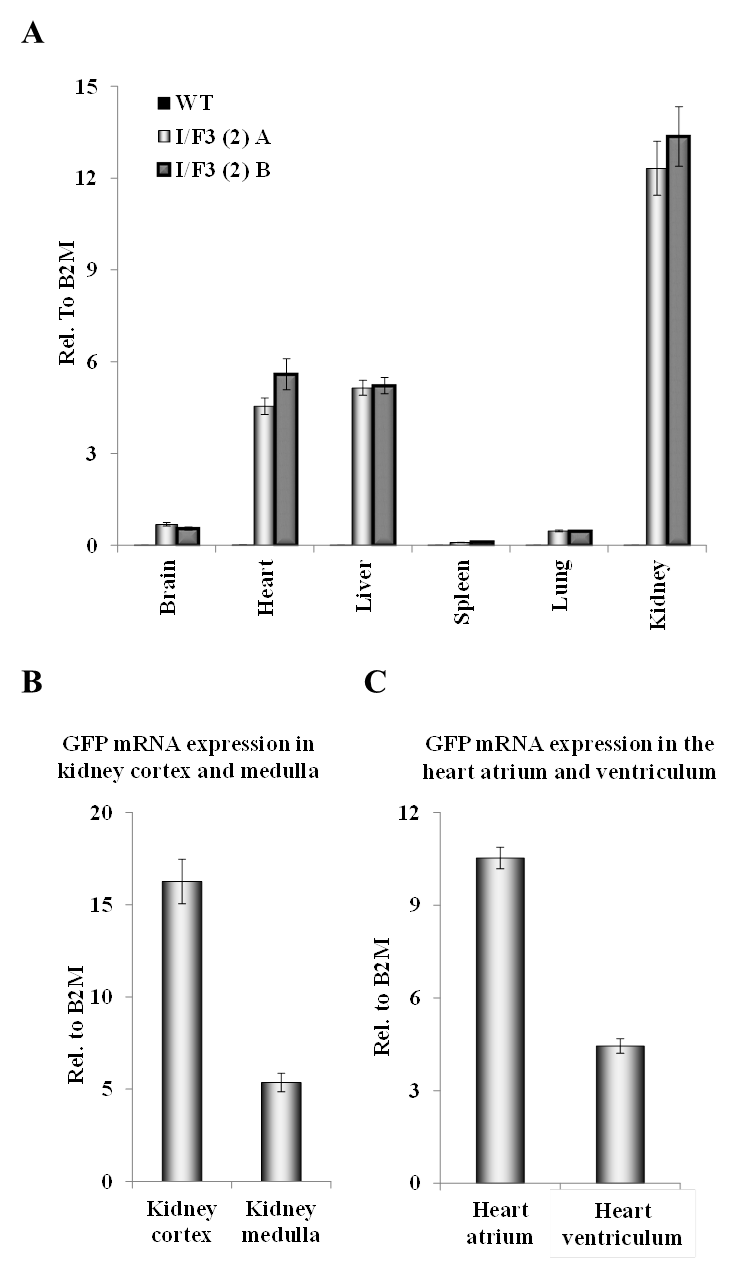


***Supplemental Figure 4***.

**Detection of GCaMP2 mRNA expression by quantitative real-time PCR measurements***.* A, GCaMP2 mRNA expression in wild type and two transgenic samples (A and B) prepared from whole brain, heart, liver, spleen, lung, and kidney. Samples indicated by A and B were obtained from two different animals born in the same litter. B, GCaMP2 mRNA expression in transgenic samples from renal cortex and medulla. C, GCaMP2 mRNA expression in transgenic samples from heart atrium and ventricle. Representative measurements on pooled samples are shown, indicating relative expression mean values ± standard deviation.

***Supplemental Figure 5***.

**Characterization of transgenic atrial and ventricular cell cultures.** Immunostaining against cardiac Troponin T (cTnT) shows cardiomyocyte-rich regions in the established primary atrial and ventricular cell cultures. Na^+^/Ca^2+^ exchanger (NCX) expression in atrial and ventricular cell cultures. Cultures generated from ventricular (upper row) or atrial (lower row) tissues were fixed and stained for NCX protein with the R3F1 antibody (kind gift of Kenneth Philipson and Michaela Ottilia, UCLA, USA). Expression patterns were somewhat different in the two cultures, as atrial cells showed high level NCX expression in the cell membrane, while ventricular cells were less efficiently stained. Also, in addition to membrane staining, intracellular staining was also observed. Nuclei were stained with DAPI.

***Supplemental Figure 6***.

**The effect of terodiline- and terfenadine on ligand-induced alteration of calcium signals *in vitro*.** Amplitude (F/F0) values of either terodiline (10 µM) or terfenadine (6.0 µM) pre-treated calcium transients evoked by adrenalin or ATP are compared to the control (calcium transients evoked by adrenalin or ATP in cell cultures without pre-treatment) in TG ventricular cell cultures. Ionomycin and EGTA were used for calibration purposes. Ventricular cultures (isolated from right and left ventricular walls) were established from three different transgenic rats, the data represents mean values ± S.E.M.


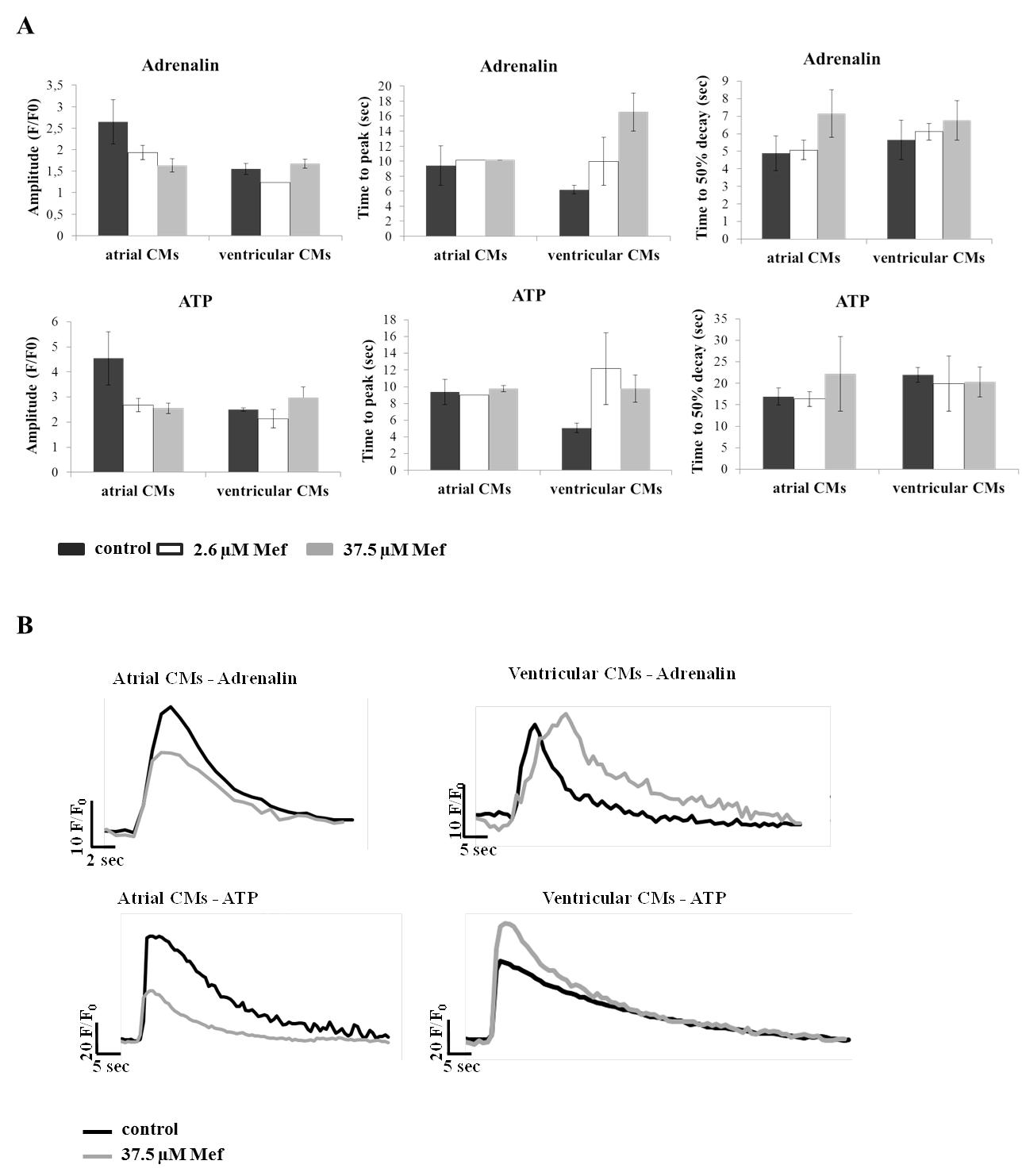


***Supplemental Figure 7***.

**Comparison of ligand-induced calcium signals of control and mefloquine pretreated cardiomyocyte cultures.** A**,** Amplitude (F/F0), time to peak (sec) and time to 50% decay (sec) values of calcium transients in atrial (atrial CMs) and ventricular cell cultures (ventricular CMs) obtained from the heart of TG rats. Prior to administration of adrenalin and ATP the cell cultures were either pre-treated with 37.5 µM mefloquine for 100 sec (37.5 µM Mef) or with 2.6 µM mefloquine for 24 hours (24h Mef), and the evoked calcium transients were compared to controls without pretreatment. Ionomycin and EGTA were used for calibration purposes. Atrial (isolated from right and left atrium) and ventricular cultures (isolated from right and left ventricular walls) were established from three different transgenic rats, the data represents mean values ± S.E.M. B, 37.5 µM mefloquine has a different effect in atrial (left panel) and in ventricular (right panel) cultures on physiologically relevant ligand-induced calcium signals (upper row: adrenalin-induced Ca^2+^ signals, lower row: ATP-induced Ca^2+^ signals).

**Supplemental Movie Legends**

***Supplemental Movie 1.***

**Ionomycin-induced calcium signal in a CAG-GCaMP2 expressing ventricular slice.** 10 µM ionomycin was added to the tissue slice to assess functional expression of the GCaMP2 protein. Fluorescence changes were detected by confocal microscopy as described in the Methods section.
